# Survival analysis of local excision vs total mesorectal excision for middle and low rectal cancer in pT1/pT2 stage and intermediate pathological risk

**DOI:** 10.1186/s12957-019-1763-9

**Published:** 2019-12-09

**Authors:** I-Li Lai, Jeng-Fu You, Yih-Jong Chern, Wen-Sy Tsai, Jy-Ming Chiang, Pao-Shiu Hsieh, Hsin-Yuan Hung, Chien-Yuh Yeh, Sum-Fu Chiang, Cheng-Chou Lai, Rei-Ping Tang, Jinn-Shiun Chen, Yu-Jen Hsu

**Affiliations:** Division of Colon and Rectal Surgery, Chang Gung Memorial Hospital, Chang Gung University College of Medicine, Linkou, 5, Fu-Hsing Street, Guei-Shan, Tao-Yuan, Taiwan

**Keywords:** Rectal cancer, Local excision, Total mesorectal excision, Chemoradiation, Sphincter-sparing surgery

## Abstract

**Background:**

Local excision (LE) is a feasible treatment approach for rectal cancers in stage pT1 and presents low pathological risk, whereas total mesorectal excision (TME) is a reasonable treatment for more advanced cancers. On the basis of the pathology findings, surgeons may suggest TME for patients receiving LE. This study compared the survival outcomes between LE with/without chemoradiation and TME in mid and low rectal cancer patients in stage pT1/pT2, with highly selective intermediate pathological risk.

**Methods:**

This retrospective study included 134 patients who received TME and 39 patients who underwent LE for the treatment of intermediate risk (pT1 with poor differentiation, lymphovascular invasion, perineural invasion, relatively large tumor, or small-sized pT2 tumor) rectal cancer between 1998 and 2016.

**Results:**

Overall survival (OS), disease-free survival (DFS), and cumulative recurrence rate (CRR) were similar between the LE (3-year DFS 92%) and TME (3-year DFS 91%) groups. Following subgrouping into an LE with adjuvant therapy group and a TME without adjuvant therapy group, the compared survival outcomes (OS, DFS, and CRR) were found not to be statistically different. The temporary and permanent ostomy rates were higher in the TME group than in the LE group (*p* < 0.001). Rates of early and late morbidity following surgery were higher in the TME group (*p* = 0.005), and LE had similar survival compared with TME.

**Conclusion:**

For patients who had mid and low rectal cancer in stage pT1/pT2 and intermediate pathological risk, LE with chemoradiation presents an alternative treatment option for selected patients.

## Background

Total mesorectal excision (TME) is the standard surgical treatment for resectable mid and low rectal adenocarcinomas. The treatment decision for patients with mid and low rectal cancer is crucial for considerations related to quality of life (QoL). TME may cause high operative morbidity, including fecal incontinence, urinary incontinence or retention, high permanent stoma rate, and sexual dysfunction [1–5].

Local excision (LE), including transanal excision, transanal polypectomy, and transanal endoscopic microsurgery, might be able to improve the QoL of patients by achieving similar oncological outcomes. Currently, a number of studies have and continue to focus on this issue [6, 7]. Despite its advantages, LE cannot achieve TME or provide pathological node (N) status. Therefore, LE was selected for patients indicating both clinically and pathologically low risk factors. Recently, the development of adjuvant concurrent chemoradiation (CCRT) has shown improvement in oncological outcomes, with more studies suggesting LE with adjuvant CCRT as an option for selected patients. However, debates involving TME or LE as options remain controversial [8–15]. According to current National Comprehensive Cancer Network (NCCN) 2018 guidelines, patients receiving LE in stage pT1 and without high-risk features can be followed-up via observation. High-risk features include positive margins, lymphovascular invasion, poorly differentiated tumors, and submucosal invasion to the lower third of the submucosal level (SM3 invasion). High-risk pathological features and pT2 status are generally indicative of the need for TME.

An ongoing randomized-control trial (TESAR trial [16], rectal preservation treatment for early rectal cancer) attempted to resolve the controversy pertaining to types of treatment. The trial included participants receiving LE and indicated stage pT1/pT2 and intermediate pathological risk as factors for receiving this treatment. Participants were categorized into three groups: low risk, intermediate risk, and high risk. In the intermediate risk group, after receiving LE, patients were randomly assigned to the concurrent chemoradiation therapy (CCRT) or TME group. The TESAR trial intended to demonstrate LE with CCRT as non-inferior to TME in the intermediate risk group [16].

Using the careful selection method adopted in the trial, our study aimed to demonstrate LE with CCRT as oncologically equivalent to TME for mid and low rectal cancer in stage pT1/pT2, with intermediate pathological risk. Our findings pertaining to a 20-year period of therapeutic outcomes were shared at a tertiary medical center.

## Methods

### Patients and materials

**Fig. 1:**
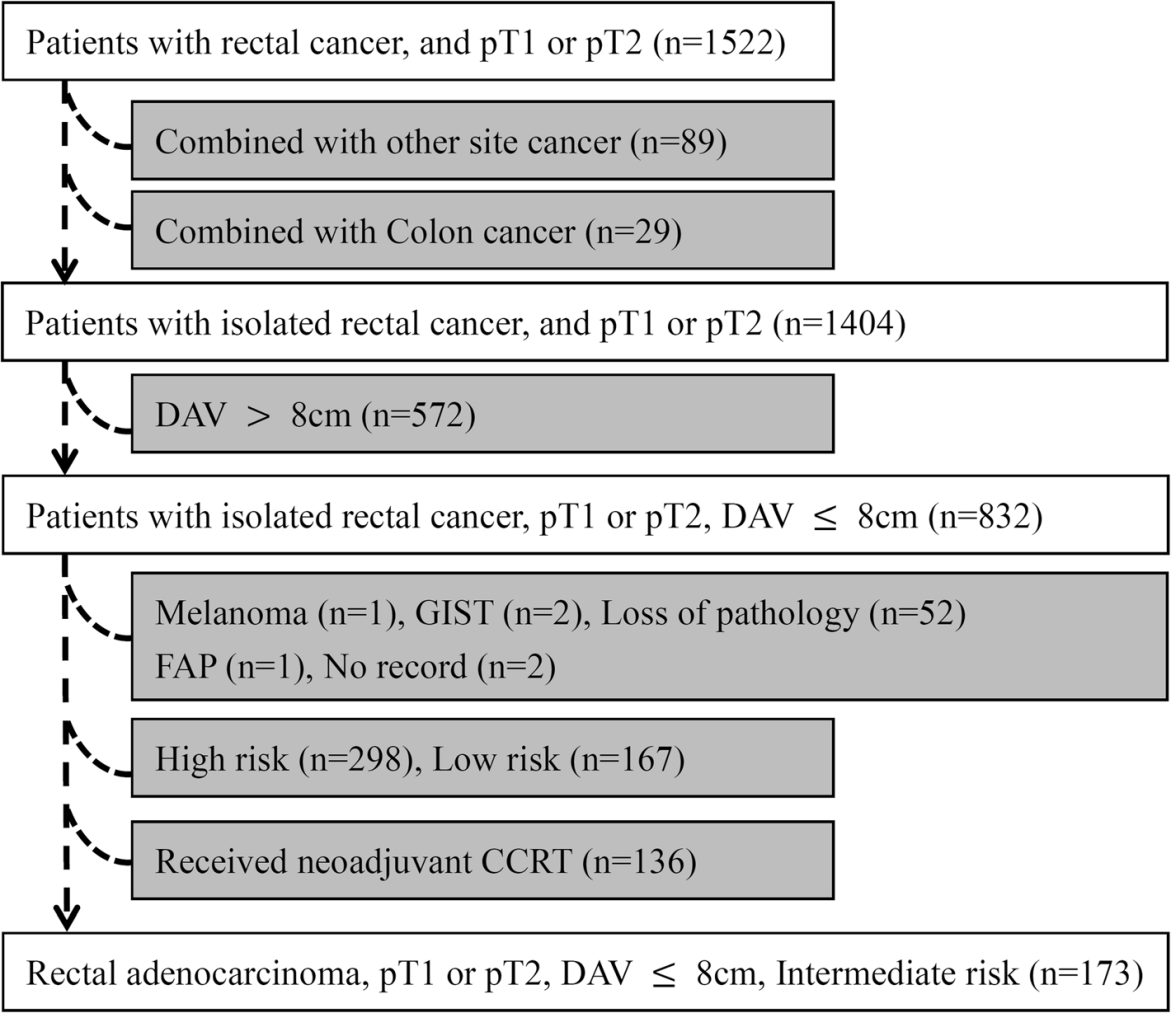
Flow chart of patient selection

Detailed data of 1522 patients who were diagnosed with pT1 or pT2 stage rectal adenocarcinoma and treated by LE or TME between January 1998 and December 2016 were retrospectively retrieved from the Colorectal Section Tumor Registry at Chang Gung Memorial Hospital, Taiwan. This study and its protocols were approved by the institutional review board of the Taoyuan branch of Chang Gung Memorial Hospital (105-1130D). Clinical staging was determined using computed tomography (CT), magnetic resonance imaging (MRI), or positron emission tomography. Patients were excluded from this study for the following reasons: clinical evidence of distant metastases, occurrence of a tumor 8 cm above the anal verge (*n* = 572), occurrence of any synchronous, and/or metachronous cancer (*n* = 118), and neo-adjuvant CCRT (*n* = 136) for rectal cancer (Fig. 1).

**Fig. 2:**
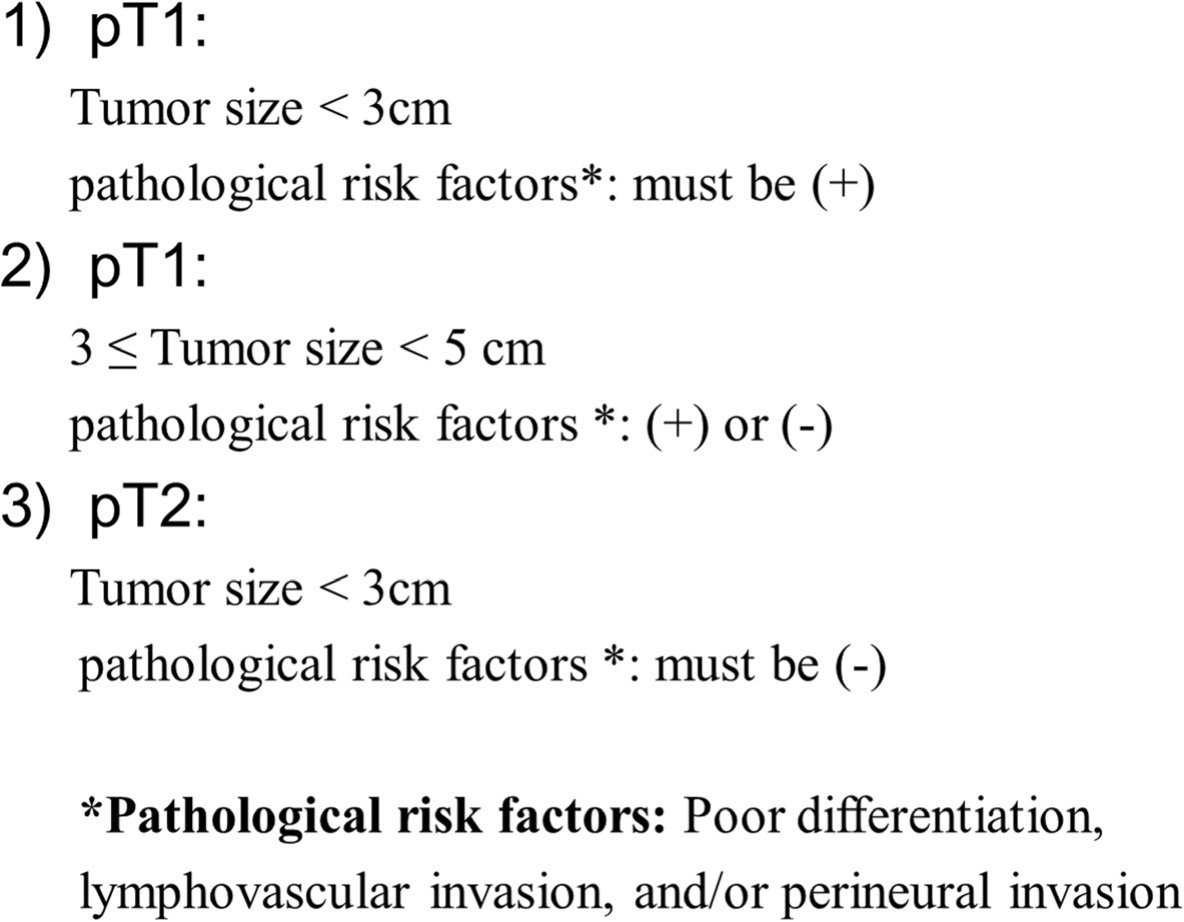
Inclusion criteria

Among the remaining patients, 173 who met any one of the following three criteria were enrolled in this study: (1) pT1 tumor < 3 cm in size and pathology exhibiting one or more risk factors, including poor differentiation and/or lymphovascular invasion (LVI) and/or perineural invasion (PNI); (2) pT1 tumor ≥ 3 cm and < 5 cm in size, with or without any pathological risk factors; and (3) pT2 tumor < 3 cm in size and pathology revealing no evidence of poor differentiation, LVI, or PNI (Fig. 2).

Patients were divided into either LE or TME group according to surgical strategies, where LE included colonoscopic polypectomy, endoscopic mucosal resection (EMR), and transanal excision (TAE) via various transanal platforms. On the other hand, TME referred to radical trans-abdominal resection, including low anterior resection (LAR) and abdominoperineal resection (APR).

The available medical records included data pertaining to age, sex, body mass index, family cancer history, tumor location (centimeters away from the anal verge), maximal tumor diameter, preoperative carcinoembryonic antigen (CEA), albumin level, and hemoglobin level. All preoperative laboratory parameters were measured within 24 h following admission. Pathological reports, including resection margin, pT and pN stage, tumor differentiation, LVI, and PNI, were examined by a specialized pathologist. Operative records included the surgery method and the creation of a temporary or permanent ostomy. Adjuvant therapy included chemotherapy or CCRT. Several chemotherapy regimens were adopted, including an oral form combining tegafur and uracil, intravenous fluorouracil (5-FU) and leucovorin, and oxaliplatin plus intravenous 5-Fu/LV (FOLFOX). Adjuvant radiotherapy with long-course radiotherapy (5040 cGy, delivered in 28 fractions) was implemented in the case of CCRT.

Postoperative complications were classified as early and late morbidity. Early morbidity was defined as postoperative complications occurring within 30 days, including wound-related (wound infection or wound dehiscence), pulmonary (atelectasis or pneumonia), cardiovascular (myocardial infarction, stroke, or embolism), urinary (urinary tract infection or neurogenic bladder), gastrointestinal (ileum obstruction or bleeding), and anastomosis-related (leakage or stenosis) complications. Late morbidity was defined as complications occurring after discharge and any event of readmission. Postoperative mortality was defined as death occurring within 30 days following surgery.

A number of physicians in the same department of this institute adopted similar follow-up routines. At the discretion of an individual physician, all patients were subjected to a follow-up program that included outpatient visits every 3 months with physical examinations, including digital rectal exams, CEA tests, CT or MRI scans, and colonoscopy. Recurrent disease was confirmed by histology of colonoscopy biopsy specimens, reoperation, or radiological studies. Prognoses were evaluated based on disease-free survival (DFS) and overall survival (OS). The DFS interval was defined as the duration between the date of initial surgery and the date of confirmation of recurrence or date of death. The OS interval was defined as the duration between the date of initial surgery and date of death. The CRR was referred to as the cumulative probability of recurring rectal cancer during follow-up.

### Statistical analysis

All analyses were performed using the Statistical Package for the Social Sciences (statistics software, version 24, IBM). Clinicopathologic characteristics were compared using the chi-square test for categorical variables and Student’s *t* test for continuous data. Cumulative recurrence rate, DFS, and OS were computed using univariate analyses according to the Kaplan–Meier method. Differences were estimated using the log-rank test. Statistical significance was set at *p* < 0.05.

## Results

Of the 173 intermediate risk rectal cancer patients, 134 received TME, and 39 received LE of a tumor. Among the 39 LE patients, 10 patients received transanal endoscopic microsurgery (TEM), three patients received EMR via colonoscopy, and 26 patients received TAE. Among the 134 TME patients, 124 patients received LAR, and 10 patients received APR. Seven patients initially received LE (three EMR and four TAE), but received TME within 1 month postoperatively because of pathology-proven cancer or because the resection margin is positive or non-evaluable.

The mean age of patients was 62.3 years. The demographic data for the TME and LE groups are shown in Table 1. No statistical difference was detected based on age, BMI, gender, cancer history of family members, preoperative CEA level, or preoperative hypoalbuminemia or anemia between groups. The temporary and permanent ostomy rates were both significantly higher in the TME group than in the LE group. The LE group had a significantly higher rate of receiving adjuvant therapy compared with the TME group (TME vs LE, 15.7% vs 43.6%, *p* < 0.001). The TME group had significantly higher early and late postoperative morbidity than the LE group. One patient in the TME group died following surgery from heart failure and sepsis because of underlying cirrhosis.

**Table 1:** Patient characteristics

Variable	TME (*N* = 134) number (%)	LE (*N* = 39) number (%)	*P* value
Age (y/o)	63.0 ± 12.9	59.7 ± 13.9	0.178
BMI (kg/m2)	24.3 ± 3.5	25.3 ± 4.4	0.226
Sex			1.000
Male	68 (50.7)	20 (51.3)	
Female	66 (49.3)	19 (48.7)	
Family cancer history	43 (32.1)	16 (41.0)	0.339
CEA (ng/mL)	2.88 ± 3.5	2.14 ± 1.1	0.757
Albumin < 3.5 (g/dL)	8 (6.0)	1 (2.9)	0.688
Hemoglobin < 10 (g/dL)	7 (5.2)	1 (2.6)	0.685
Ostomy			< 0.001
No	69 (51.5)	36 (92.3)	
Temporary	50 (37.3)	1 (2.6)	
Permanent	15 (11.2)	2 (5.1)	
Adjuvant therapy	21 (15.7)	17 (43.6)	< 0.001
Chemotherapy	*21 (15.7)	**1 (2.6)	
CCRT	0 (0)	**16 (41.0)	
Post-op morbidity	38 (28.4)	2 (5.1)	0.002
Early	25 (18.7)	2 (5.1)	0.045
Late	22 (16.4)	0 (0)	0.005

*TME* total mesorectal excision, *LE* local excision, *BMI* body mass index, *CEA* carcinoembryonic antigen, *CCRT* concurrent chemoradiation

*Including tegafur and uracil in 17 patients, intravenous form 5-FU and leucovorin in 3 patients, and oxaliplatin plus intravenous 5-Fu/LV (FOLFOX) for 6 months in 1 patient

**Including tegafur and uracil in 1 patient, tegafur, and uracil with long-course radiotherapy in 14 patients, and 5-FU/LV with long-course radiotherapy in 2 patients

Pathological data are shown in Table 2. The mean maximal diameter of tumor was not significantly different between the two groups. The distance of the tumor from the anal verge in the LE group was shorter than that in the TME group (TME vs LE, 5.93 vs 4.59 cm, *p* < 0.001). The resection margin in the LE group was significantly closer than that in the TME group (TME vs LE, 1.54 vs 0.14 cm, *p* < 0.001). Lymph node yield in the TME group was on average 16.5 (10.0–26.3). Histological differentiation and the presence of LVI and PNI were similar in the two groups. The proportion of T2 tumors was significantly higher in the TME group compared with the LE group (*p* < 0.001). In the TME group, the N1 rate was 14.2%, and the N2 rate was 2.2%.

**Table 2:** Pathological data

Variable	TME (*n* = 134) number (%)	LE (*n* = 39) number (%)	*P* value
Maximal diameter of tumor (cm)	2.55 ± 0.75	2.75 ± 0.98	0.193
Tumor distance from anal verge (cm)	5.93 ± 1.83	4.59 ± 1.92	< 0.001
Resection margin (cm)	1.54 ± 0.98	0.14 ± 0.25	< 0.001
Lymph nodes yield*	16.5 [10.0-26.3]	-	-
Differentiation			0.427
Well	31 (23.1)	13 (33.3)	
Moderate	98 (73.1)	25 (64.1)	
Poor	5 (3.7)	1 (2.6)	
Lymphovascular invasion	11 (8.2)	7 (17.9)	0.131
Perineural invasion	4 (3.0)	0 (0)	0.576
T stage			< 0.001
T1	48 (35.8)	31 (79.5)	
T2	86 (64.2)	8 (20.5)	
N stage			**-**
N0	112 (83.6)	-	
N1	19 (14.2)	-	
N2	3 (2.2)	-	

*TME* total mesorectal excision, *LE* local excision

*Median [1st quartile–3rd quartile]

**Fig. 3:**
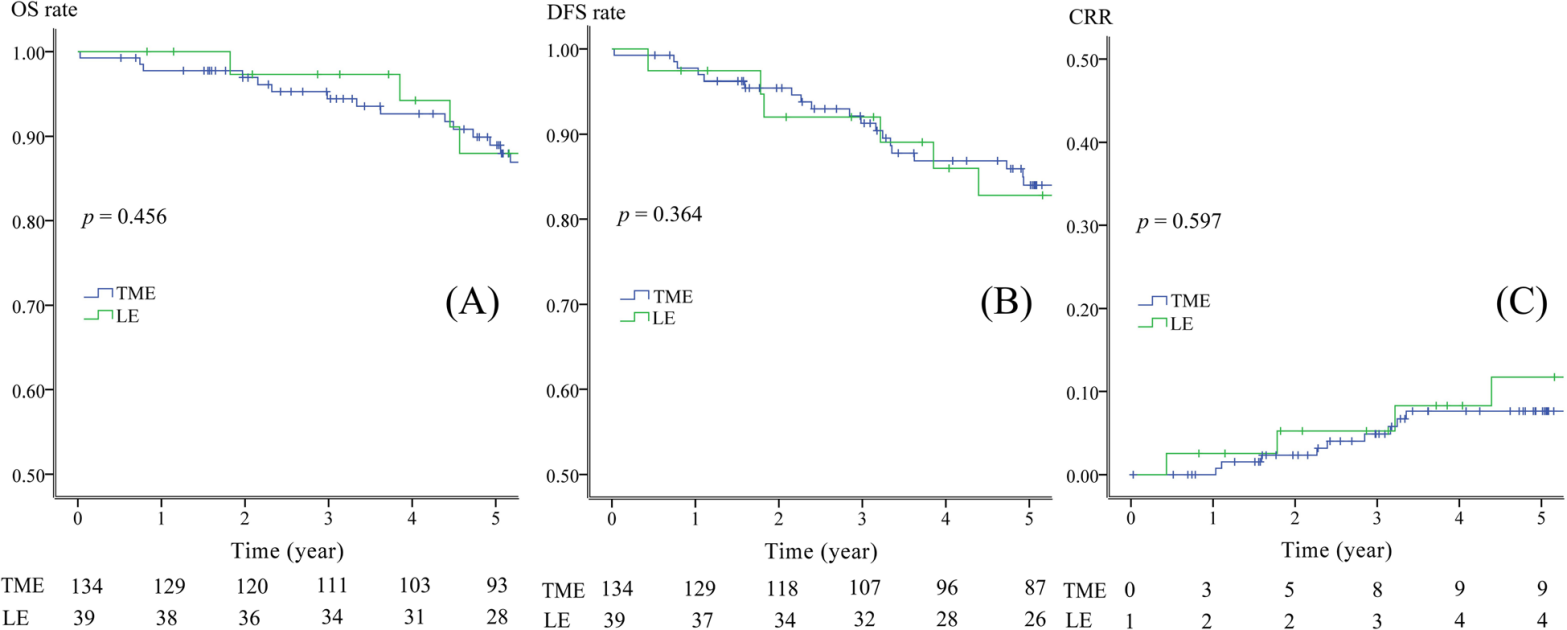
Survival analyses on all 173 patients (OS, DFS, and CRR). **a** OS of 134 patients received TME vs. 39 patients received LE. **b** DFS of 134 patients received TME vs. 39 patients received LE. **c** CRR of 134 patients received TME vs. 39 patients received LE

The median follow-up time was 96.3 months in the TME group and 73.6 months in the LE group. Fourteen patients (8.1%) were found to exhibit six local recurrence events and 12 distant metastases events in total (Table 3). The estimated OS, DFS, and CR rates are shown in Fig. 3. The survival rates are listed in 1-year, 3-year, and 5-year blocks, respectively (Table 3). No survival difference was observed between the TME and LE groups.

**Table 3:** Recurrence status and the prediction of survival in TME or LE

Variable	TME (*n* = 134)	LE (*n* = 39)	*P* value
Recurrence (events)			
Local recurrence	3 (2.2%)	3 (7.7%)	0.104
Distant metastasis	9 (6.7%)	3 (7.7%)	0.846
Overall survival (OS)			0.456
1-year	98%	100%	
3-year	94%	97%	
5-year	89%	88%	
Disease-free survival (DFS)			0.364
1-year	98%	97%	
3-year	91%	92%	
5-year	84%	83%	
Cumulative recurrence rate (CRR)			0.597
1-year	0%	3%	
3-year	5%	5%	
5-year	8%	12%	

*TME* total mesorectal excision, *LE* local excision

**Fig. 4:**
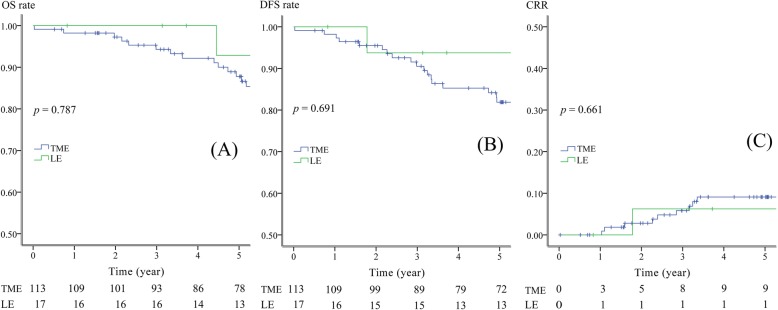
Survival analyses with subgrouping. **a** OS of 113 patients received TME without adjuvant therapy vs. 17 patients received LE with adjuvant therapy. **b** DFS of 113 patients received TME without adjuvant therapy vs. 17 patients received LE with adjuvant therapy. **c** CRR of 113 patients received TME without adjuvant therapy vs. 17 patients received LE with adjuvant therapy

In additional comparisons, we distributed 113 patients into a TME without adjuvant therapy group and 17 patients into an LE with adjuvant therapy group. There was one N1 patient in the TME without adjuvant therapy group and no N2 patient. The estimated 3-year OS rate was 94% in the TME without adjuvant therapy group and 100% in the LE with adjuvant therapy group (Fig. 4a, *p* = 0.787). The estimated 3-year DFS rate was 90% in the TME without adjuvant therapy group and 94% in the LE with adjuvant therapy group (Fig. 4b, *p* = 0.691). The estimated 3-year CRR was 6% in the TME group and 6% in the LE group (Fig. 4c, *p* = 0.661).

## Discussion

We grouped our early stage rectal cancer patients into three groups: low risk, intermediate risk, and high risk. As is current practice, we generally arranged LE for low risk patients. In a review of current studies, no studies were found comparing the oncological outcomes of patients with intermediate risk between LE and TME. Our study attempted to conclude that if a pathological intermediate risk rectal cancer remained after curative LE, chemoradiation may be an alternative treatment option.

### Concurrent chemoradiation

Perioperative radiotherapy downsizes or downstages the primary tumor and reduces the risk of intraoperative tumor seeding [17]. Some patients may even achieve complete cancer resolution. Regarding the possibility of resolution, we excluded patients receiving neo-adjuvant long-course or short-course radiotherapy.

By subgrouping patients into a TME without adjuvant therapy group and an LE with adjuvant therapy group, we observed that these two groups had similar oncological outcomes. Among the 113 cases selected for TME without adjuvant therapy, 10 experienced cancer recurrence. One of the 10 patients had a pN1b status. The patient refused adjuvant therapy because of old age and experienced brain metastasis 3 years following LAR. Among the 17 patients who received LE and adjuvant therapy, one had lung metastasis postoperatively at 21 months. Among the remaining 22 patients who received LE without adjuvant therapy, three had local recurrence postoperatively at 6, 38, and 52 months (Table 4).

**Table 4:** Local recurrence and distant metastasis in TME or LE

Patients	Sex	Age	Operation	Adjuvant therapy	Local recurrence	Distant metastasis	DAV (cm)	Tumor diameter (cm)	T stage	N stage	Resection margin (cm)
LE 1	F	51	TEM	No	Excision site	Lung	5	4.0	T1	n/a	0.1
LE 2	M	31	TEM	CCRT	No	Lung	5	1.0	T1	n/a	0.1
LE 3	F	74	Polypectomy	No	Excision site	Lung	5	3.8	T1	n/a	< 0.1
LE 4	F	26	TRE	No	Excision site	No	3	4.0	T1	n/a	< 0.1
TME 1	M	58	LAR	No	Anastomosis	Liver	3	3.2	T1	N0	0.5
TME 2	F	49	LAR	No	No	PA nodes	8	2.0	T2	N0	1.7
TME 3	M	54	LAR	No	Anastomosis	No	4	2.0	T2	N0	0.7
TME 4	M	71	LAR	No	Presacral	Lung	6	2.0	T2	N0	1.0
TME 5	F	68	LAR	No	No	Lung	5	2.0	T2	N0	0.7
TME 6	F	63	LAR	No	No	Lung	8	2.7	T2	N0	2.0
TME 7	F	55	APR	No	No	Lung	3	2.5	T2	N0	3.0
TME 8	M	75	APR	No	No	Lung	3	2.4	T2	N0	3.0
TME 9	M	76	LAR	No	No	Brain	5	2.1	T2	N1b	0.6
TME 10	M	72	APR	No	No	Lung	2.5	4.5	T1	N0	2.5

*APR* Abdomino-perineal resection, *CCRT* concurrent chemoradiation, *DAV* distance from anal verge, *LAR* low anterior resection, *LE* local excision, *PA* para-aortic, *TEM* trans-anal endoscopic surgery, *TME* total mesorectal excision, *TRE* trans-rectal excision

Preoperative or postoperative CCRT can give rise to a number of side effects. Several studies have reported side effects such as diarrhea, proctitis, fistula, perforation, and permanent incontinence. The complication rate ranged from 6.5 to 52% [17–19]. In our study, 17 of 39 patients received LE with CCRT. Long-course radiotherapy (5040 cGy, delivered in 28 fractions) was implemented in 16 cases. No radiotherapy-related complications or morbidity was noted.

### T stage, resection margin, and recurrence

**Fig. 5:**
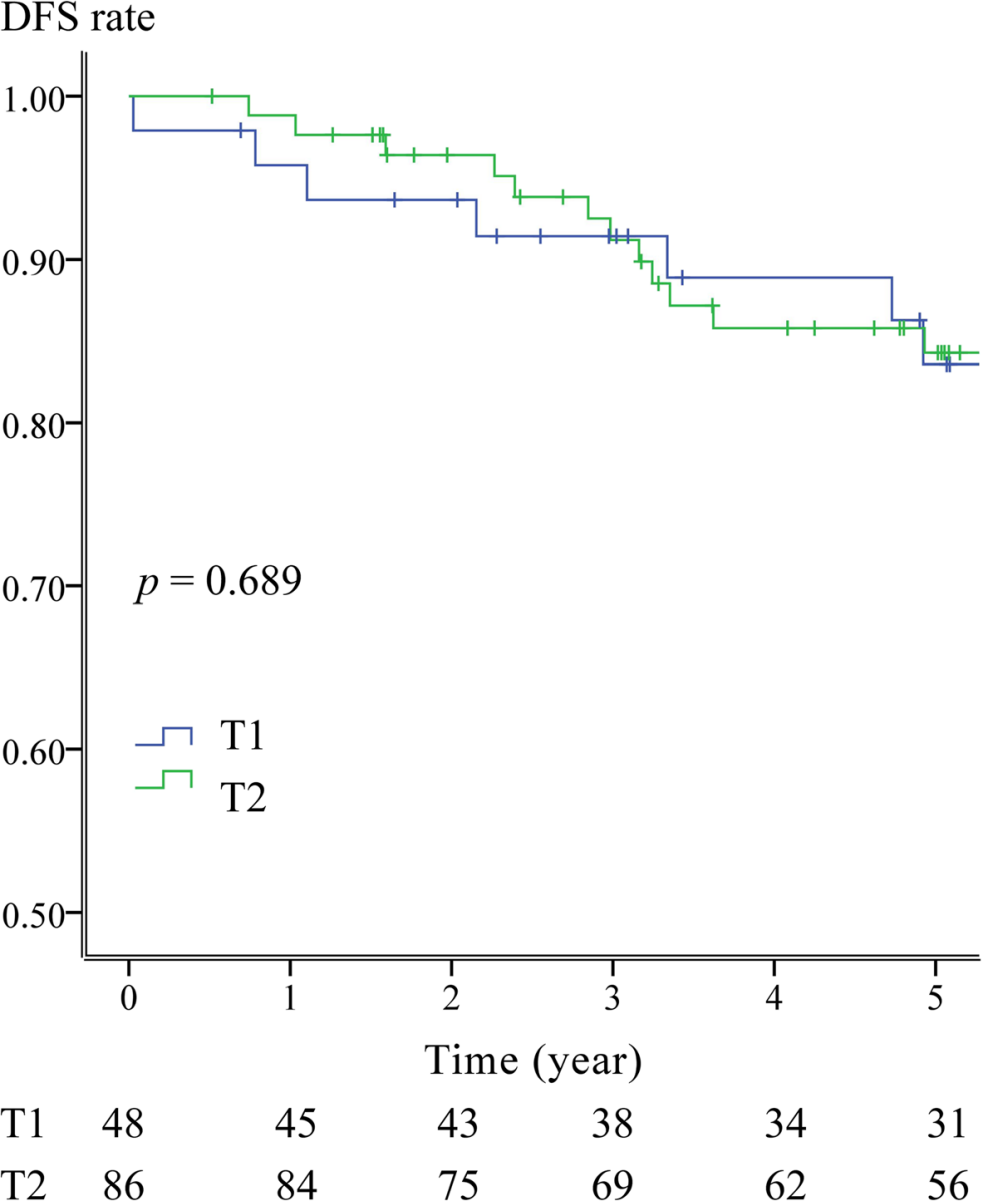
DFS of T1 vs T2 in 134 patients received TME. This figure illustrated the DFS of T1 (48 cases) vs. T2 (86 cases). By standardizing the treatment effect, we selected only patient received TME

In our study, the TME and LE groups had different compositions regarding T stage distribution (Table 4). To our knowledge, T stage is a predisposing factor and presents a high risk for the presence of a T2 tumor. However, with the evolution of transanal microsurgery and adjuvant therapy, more studies suggest LE for selected T2 patients [20–24]. According to the selection criteria in this study, patients with T1 tumors indicated a larger tumor size or higher histological risk than patients with T2 tumors. In order to standardize the therapeutic effect, we focused only on the 134 patients who received TME and conducted additional survival analysis. In this analysis, we found that patients with T1 or T2 tumors showed no significant difference regarding disease-free survival (Fig. 5, *p* = 0.689), local-recurrence-free survival (*p* = 0.976), or distant metastasis-free survival (*p* = 0.432).

Resection margin is a predisposing factor for postoperative local recurrence in rectal cancer [25–28]. A 0.3-cm clear margin was acceptable for LE, and a 1 to 2-cm distal resection margin was adequate for TME [29]. Despite a systematic review drawing conclusions against the 1-cm rule, a 1-cm resection margin still had borderline significance (*p* = 0.09) on five-year local recurrence [30]. In our study, patients who experienced local recurrence had a lower resection margin (0.1 cm or closer in the LE group; 0.5 to 1.0 cm in the TME group). Furthermore, patients who received LE had higher local recurrence rates than those who received TME (7.7% vs 2.2%, *p* = 0.104). A retrospective study showed similar findings, i.e., that a resection margin ≤ 0.1 cm correlated with local recurrence in pT1 rectal cancers following TEM [27].

### Local recurrence and distant metastasis in LE and TME

Early stage cancer recurrence is frustrating to both the patient and the surgeon. In stage I rectal cancer, the 5-year local recurrence rates ranged from 4 to 20% after LE and from 0 to 10% after TME [31, 32]. In our study, an insufficient resection margin may be the reason why the LE group had a higher local recurrence rate than the TME group. No statistical difference in distant metastasis was found between the LE and TME groups. Local recurrence found in the pelvic cavity or endoluminally should be treated with radical salvage resection [33]. In our study, local recurrences were treated with salvage LAR or APR. Among the three local recurrences after LE, two of them had local recurrence postoperatively after 6 months and 52 months. Both of them received salvage APR. One of them experienced lung metastasis postoperatively after 43 months, while another one died from other cause after 3 years follow-up. The last one of the three patients was detected with local recurrence postoperatively after 38 months and lung metastasis almost at the same time.

Additionally, LE with neo-adjuvant CCRT or adjuvant CCRT/radiotherapy may decrease local recurrence rate [20, 21, 23, 24]. In our study, 17 patients who received LE followed by CCRT had no local recurrence. It is worth noting that if local recurrence still occurs, CCRT may increase the difficulty of salvage resection and give rise to a higher R1 resection rate [34].

### Postoperative morbidity and prognosis

LE causes lower morbidity (e.g., a sphincter-sparing procedure), lower mortality, and rapid postoperative recovery. The colostomy rate, perioperative mortality/complications, and low anterior resection syndrome (LARS) rate in the LE group were relatively low, but the local recurrence rate was higher [2, 4, 13]. The symptoms of LARS (including fecal incontinence or urgency, frequent or fragmented bowel movements, emptying difficulties, and increased intestinal gas) have an immense impact on patients’ QoL. Dr. Nancy You drew the same conclusion by utilizing the National Cancer Database (NCDB) [35]. In our study, the LE group had a lower postoperative morbidity rate (5.1% vs 28.4%) and permanent ostomy rate (5.1% vs 11.2%), compared with the TME group.

## Limitations

There were several potential limitations to this study. First, the study was limited by its retrospective design and limited sample size, which may have given rise to inherent biases among patients or in treatment characteristics in a long-term follow-up study. Second, owing to this retrospective nature, the presence of symptoms, severity of disease, comorbidity, and patient preference may have altered treatment choice, thereby contributing to patient selection bias. Third, patients were assigned to the LE or TME group based on CTs/MRIs, colonoscopy, and digital rectal examination. The distribution of pT1/pT2, for example, was different in both groups. Though pT2 tumors were considered to lead to inferior outcomes, pT1/pT2 tumors were considered to pose similar risks in our study because of the specific patient selection criteria.

## Conclusions

In conclusion, LE with adjuvant chemoradiation is an alternative treatment option for mid and low early stage rectal cancer with intermediate pathological risk (poor differentiation, lymphovascular invasion, perineural invasion, relatively large tumor, and T2 tumor). Long-term survival outcomes were not statistically different between patients who received LE and those who received TME. Patients who received LE had lower postoperative incidence of morbidity and temporary or permanent ostomy.

## Data Availability

The detailed patients’ databases generated and analyzed during this study are not publicly available due to appropriate protection of patients’ personal information but are available from the corresponding author on reasonable request.

## Abbreviations

APR: Abdominoperineal resection
BMI: Body mass index
CCRT: Concurrent chemoradiation
CEA: Carcinoembryonic antigen CRR Cumulative recurrence rate
CT: Computed tomography
DFS: Disease-free survival
EMR: Endoscopic mucosal resection
LAR: Low anterior resection
LE: Local excision
LVI: Lymphovascular invasion
OS: Overall survival
PNI: Perineural invasion
QoL: Quality of life
TAE: Transanal excision
TEM: Transanal endoscopic microsurgery
TME: Total mesorectal excision
